# Alterations in Exercise-Induced Plasma Adenosine Triphosphate Concentration in Highly Trained Athletes in a One-Year Training Cycle

**DOI:** 10.3390/metabo9100230

**Published:** 2019-10-16

**Authors:** Ewa Anna Zarębska, Krzysztof Kusy, Ewa Maria Słomińska, Łukasz Kruszyna, Jacek Zieliński

**Affiliations:** 1 Department of Athletics, Strength and Conditioning, Poznan University of Physical Education, Królowej Jadwigi 27/39, 61-871 Poznan, Poland; kusy@awf.poznan.pl; 2 Department of Biochemistry, Medical University of Gdansk, Dębinki 1, 80-211 Gdansk, Poland; eslom@gumed.edu.pl; 3 Department of General and Vascular Surgery, Poznan University of Medical Sciences, Długa 1/2, 61-848 Poznan, Poland; lukaszkruszyna@poczta.onet.pl

**Keywords:** annual training cycle, ATP release, plasma nucleotides, training adaptation, incremental exercise test

## Abstract

This study aimed to assess the effect of training loads on plasma adenosine triphosphate responsiveness in highly trained athletes in a 1 y cycle. Highly trained futsal players (11 men, age range 20–31 y), endurance athletes (11 men, age range 18–31 y), sprinters (11 men, age range 21–30 y), and control group (11 men, age range 22–34 y) were examined across four characteristic training phases in response to an incremental treadmill test until exhaustion. A considerably higher exercise and post-exercise plasma adenosine triphosphate concentrations were observed in consecutive training phases in highly trained athletes, with the highest values reached after the competitive period. No differences in plasma adenosine triphosphate concentrations were found in the control group during the 1 y cycle. Sprinters showed a higher absolute and net increase in plasma adenosine triphosphate concentration by 60–114% during exercise in consecutive training phases than futsal players (63–101%) and endurance athletes (64–95%). In this study, we demonstrated that exercise-induced adenosine triphosphate concentration significantly changes in highly trained athletes over an annual training cycle. The obtained results showed that high-intensity but not low- to moderate-intensity training leads to an increased adenosine triphosphate response to exercise, suggesting an important role of ATP for vascular plasticity.

## 1. Introduction

Exercise-related shear stress, local hemoglobin desaturation, and increased temperature in the working muscle are typical phenomena that induce adenosine triphosphate (ATP) release, predominantly from erythrocytes, to improve local blood flow [1,2,3]. The vasodilatory response during prolonged dynamic exercise is due to thermal and metabolic rate-sensing mechanisms within skeletal muscle, presumably through signaling pathways that regulate the intravascular concentration of ATP [4]. Plasma ATP levels are increased in the venous effluent from exercising muscle [5]. Intravascular ATP can independently attenuate α_1_-adrenergic vasoconstriction, which further supports the potential blood flow regulative role of ATP during exercise in humans [6,7,8]. Furthermore, ATP can elicit prolonged vasodilatation for up to 3 h [9]; therefore, it is an attractive mediating signal because of its sympatholytic and skeletal muscle blood flow regulation properties. Adenosine diphosphate (ADP), an ATP derivative, has been proposed to mediate the middle phase of reactive hyperemia via endothelial P2Y_1_ receptors [10]. ADP also activates a negative feedback pathway of ATP release from erythrocytes via P2Y_13_ receptors [11]. Therefore, an increased ADP concentration during exercise [12] may be of great physiological importance to diminishing ATP release. Plasma adenosine monophosphate (AMP), alongside from ATP and ADP, has also been shown to increase during submaximal and maximal exercise intensity [13]. However, its importance to the vasodilatory response is minor [7].

Repeated elevated shear stress improves the flow-mediated dilatation of large conduit arteries as well as enhancing vasodilatory capacity during isolated exercise in trained muscles [14]. Long-lasting physical training causes specific adaptations in response to the unique demands of different types of training, i.e., enhanced oxygen (O_2_) efflux through increased maximal cardiac output, improved blood flow resulting from vascularization, and improved erythrocyte deformability [15]. It appears that high-volume, low-intensity training is crucial to providing a platform for specific adaptations that are developed in response to high-intensity exercise [16]. For example, endurance training programs promote skeletal muscle capillary supply and muscle fiber oxidative capacity with little increase in either muscle strength and muscle size [17,18]. In particular, aerobically trained athletes show enhanced vasodilatory and venous capacity in their muscles [19]. Well-trained endurance athletes perform ~80% of their training at intensities below the lactate threshold (low-intensity exercise zone), despite competing at intensities reaching maximal oxygen consumption (VO_2_max) [20]. The speed–power training used by sprinters is qualitatively different. The relative anaerobic energy system contribution is estimated at around 70–80% [21]. Speed–power training promotes the maximization of speed–strength abilities relative to body mass, in addition to modest increases in both capillary supply and oxidative capacity [18]. Specific muscle adaptations to sprint training are associated with the high metabolic demands of high-intensity muscle contractions. A mixed training regime, e.g., in futsal players, involves multiple high-intensity intermittent exercise bouts during training sessions and matches, which induce significant muscle fatigue [22]. Such a specific training program is aimed at developing intermittent endurance capacity, repeated maximal sprint ability, and power maximization [23]. In the above-mentioned training profiles, most of the total body muscle mass is activated, but the stress placed on the central circulation to suddenly provide blood flow seems to be much more marked in resistance than endurance training. Futsal training requires, in turn, multiple moments of high blood flow delivery during prolonged exercise. A recent study showed the impact of sport specialization on exercise-induced plasma ([ATP]) in highly trained athletes, and indicated that total-body skeletal muscle mass is an important factor [12]. We believe there is a need for further research addressing the effect of long-term whole-body training on vascular function, and in particular, its influence on ATP and its derivatives. We presume that these results will improve the understanding of metabolic adaptation to long-term structured training programs. Possible future applications of this knowledge include applications in the fields of exercise medicine, sport, and public health.

To the best of our knowledge, no previous studies have encompassed the changes in plasma nucleotide concentration during an annual training cycle, taking into account the amount and type of training load. Thus, the plasma ATP exercise-induced response within consecutive phases of periodized endurance, speed–power, or mixed training is still unknown.

## 2. Results

### 2.1. Training Characteristics

The exercise loads between training subphases in competitive athletes were precisely monitored. Every exercise was assigned to the one of “energy zones”, simplified for the needs of this article, that corresponded with estimates of energy sources for ATP resynthesis [24]. In control subjects, the levels of training loads remained unchanged during the whole study period. They recreationally practiced running at moderate intensity 3–5 times per week. During a 1 month transition phase, competitive athletes focused on physical and psychological regeneration and recovery from injuries. The low-intensity training loads mainly consisted of activity forms other than those typical of athletes’ primary sports disciplines. The aim of the general subphase (12 weeks) was to develop general endurance based on low-intensity training and to increase general fitness. The specific subphase lasted 12 weeks and was focused on the development of specific endurance and physical fitness, and was mostly based on high-intensity interval exercise and speed runs. The competition phase (10 weeks) was characterized by increased intensity and decreased volume of training. Athletes competed in their specialized distances, reaching peak performance. More detailed training characteristics of the consecutive examinations are presented in Table 1.

### 2.2. Pre-Exercise Nucleotide Concentration

**Figure 1:**
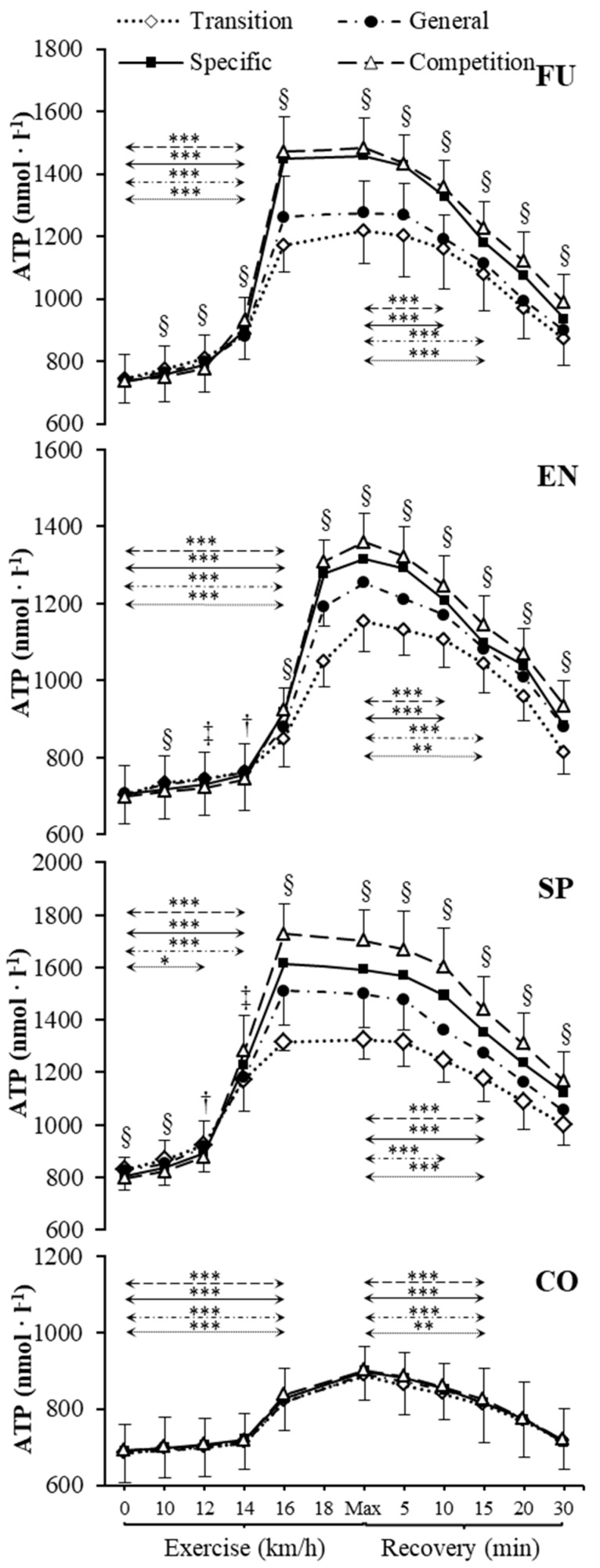
Venous plasma [ATP] before exercise, during incremental treadmill test until exhaustion, and post-exercise recovery in futsal players (FU; *n* = 11), endurance athletes (EN; *n* = 11), sprinters (SP; *n* = 11), and control group (CO; *n* = 11) in four consecutive training phases. Arrows indicate the first significant differences from samples taken at rest and maximal exercise within examinations. Significant differences between blood sampling points: *** *p* < 0.001, ** *p* < 0.01, * *p* < 0.05. Significant. differences between examinations at the same sampling point: § *p* < 0.001, ‡ *p* < 0.01, † *p* < 0.05. Data are presented as means ± SD.

**Figure 2:**
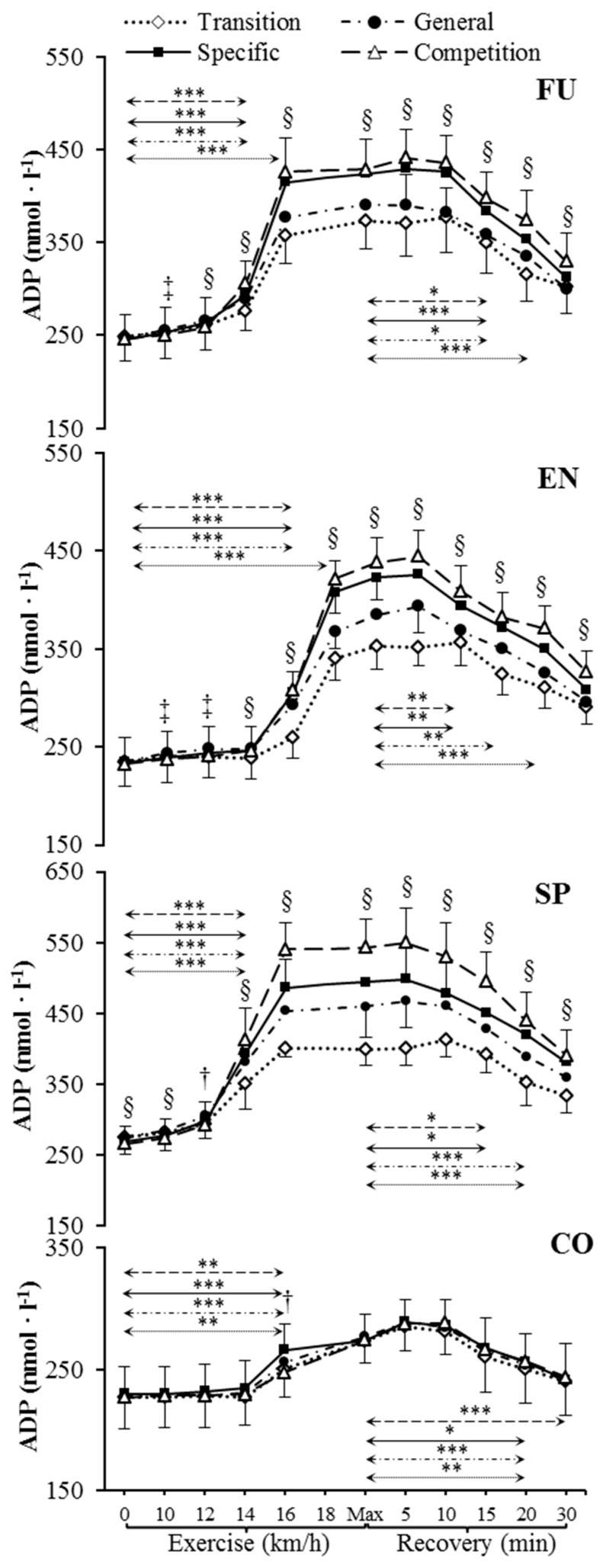
Venous plasma [ADP] before exercise, during incremental treadmill test until exhaustion, and post-exercise recovery in futsal players (FU; *n* = 11), endurance athletes (EN; *n* = 11), sprinters (SP; *n* = 11), and control group (CO; *n* = 11) in four consecutive training phases. Arrows indicate the first significant differences from samples taken at rest and maximal exercise within examinations. Significant differences between blood sampling points: *** *p* < 0.001, ** *p* < 0.01, * *p* < 0.05. Significant differences between examinations at the same sampling point: § *p* < 0.001, ‡ *p* < 0.01, † *p* < 0.05. Data are presented as means ± SD.

**Figure 3:**
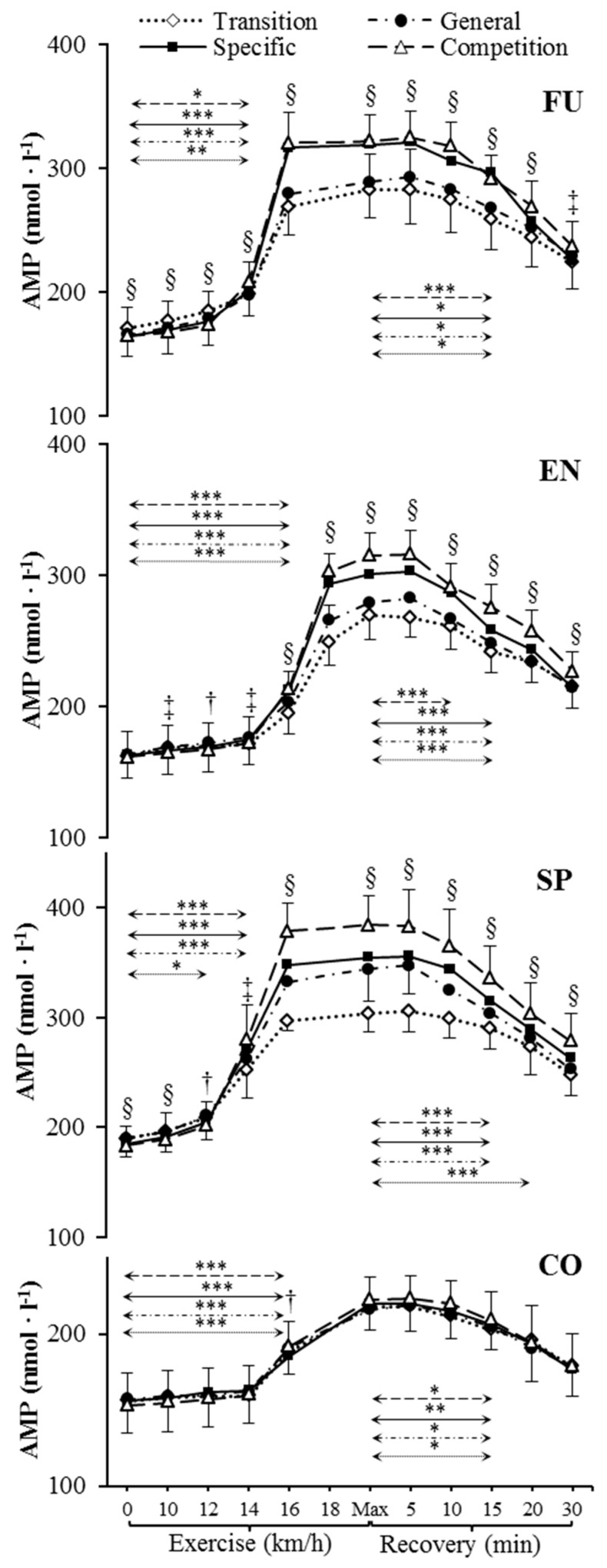
Venous plasma [AMP] before exercise, during incremental treadmill test until exhaustion, and post-exercise recovery in futsal players (FU; *n* = 11), endurance athletes (EN; *n* = 11), sprinters (SP; *n* = 11), and control group (CO; *n* = 11) in four consecutive training phases. Arrows indicate the first significant differences from samples taken at rest and maximal exercise within examinations. Significant differences between blood sampling points: *** *p* < 0.001, ** *p* < 0.01, * *p* < 0.05. Significant differences between examinations at the same sampling point: § *p* < 0.001, ‡ *p* < 0.01, † *p* < 0.05. Data are presented as means ± SD.

Resting venous plasma [ATP], [ADP] and [AMP] significantly differed between training phases in sprinters (specific preparation and competition phase differed from both transition and general preparation phase) (Figure 1, Figure 2 and Figure 3). Pre-exercise [AMP] after the transition phase differed from other phases in futsal players (Figure 3).

### 2.3. Nucleotide Concentration during Exercise

Our data showed significant increases in plasma [ATP], [ADP], and [AMP] during exercise (Figure 1, Figure 2 and Figure 3) in each athletic group throughout the whole 1 y training cycle, but not in controls. All athletes reached their peak plasma [ATP] at the end of the test, except sprinters. None of the athletic groups reached peak plasma [ADP] and [AMP] at maximum intensity at the end of the exercise. The level of ATP, ADP, and AMP during exercise was considerably higher in all competitive athletes in each consecutive examination. In sprinters, the exercise-induced [ATP] net increase above the baseline after the transition and competition phase was 60% and 114%, respectively. In futsal players, exercise [ATP] values changed significantly (*p* < 0.001), causing a net increase of 63% after the transition period and 101% after the competition period. A similar pattern of change was noted in endurance athletes; however, the change was smaller in this group. In endurance athletes, ATP concentration increased by 64% after the transition period, and 95% after the competition period.

### 2.4. Nucleotide Concentration at Maximal Intensity

[ATP], [ADP], and [AMP] at maximal exercise differed between groups in consecutive training phases (*p* < 0.001). Between all groups, except for futsal players and endurance athletes, maximal [ATP] differed after transition and general preparation phase (*p* < 0.001). After the specific preparation and competition phases, [ATP] differed between all athletic groups (*p* < 0.001). Sprinters presented higher [ATP] at maximal intensity than futsal players and endurance athletes throughout the whole training cycle. After the transition phase, maximal [ADP] and [AMP] in the control group were lower than in other groups (*p* < 0.001). Additionally, higher peak [ADP] and [AMP] were observed in sprinters than in endurance athletes (*p* < 0.001). In consecutive training phases, [ADP] and [AMP] at maximal exercise controls and sprinters varied compared to other groups (*p* < 0.001). Sprinters presented the highest maximal [ADP] and [AMP], starting from the general preparation phase.

### 2.5. Nucleotide Concentration during Recovery

During recovery, there was a significant decrease in venous plasma nucleotide concentration in competitive athletes in each training period. In controls, no significant changes in recovery plasma [ATP], [ADP], and [AMP] during the 1 y training cycle were observed. The first significant plasma [ATP], [ADP], and [AMP] decrease compared to the peak value and differences between examinations at the same sampling point are presented in Figure 1, Figure 2 and Figure 3. Additionally, [ATP], [ADP], and [AMP] values reported after 30 min of recovery were significantly different from those obtained pre-exercise in competitive athletes, but not in controls, except for [AMP].

### 2.6. Respiratory Compensation Point

Respiratory compensation point (RCP) expressed as a percentage of VO_2_max occurred within the range of 85% to 94% in all groups. As regards the running speed, the RCP occurred between 16 and 18 km·h^−1^ in endurance athletes and between 14 and 16 km·h^−1^ in sprinters in all training phases. Futsal players reached RCP at between 12 and 14 km·h^−1^ after the transition phase and general subphase of the preparatory phase. After the specific subphase of the preparatory phase and the competition phase, RCP was reached at between 14 and 16 km·h^−1^ in futsal players. In the control group, the RCP occurred between 14 and 16 km·h^−1^ during all examinations, except for the 2nd examination where RCP was reached after 16 km·h^−1^.

## 3. Discussion

This was the first study to investigate the changes in plasma nucleotide concentration in response to an annual training cycle in highly trained athletes from distinct sports disciplines. The primary novel findings were as follows: (1) exercise-induced plasma [ATP] significantly changed over the annual training cycle in highly trained athletes (increases from transition to competition phase), (2) sprint training brought about higher absolute exercise-induced plasma [ATP] than endurance and mixed training or recreational non-periodized activity, and (3) in spite of differences in magnitude, each kind of structured training program (sprint, endurance, or mixed) incorporating a sufficient amount of high-intensity exercise led to the same adaptation pattern.

The key factor seems to have been the proportion of high-intensity training loads that were related to increased plasma [ATP] in the competition period. The reduction or lack of high-intensity exercise in other training phases was associated with a decrease in plasma [ATP]. In competitive athletes, the sudden increase in plasma [ATP] during exercise was concurrent with the occurrence of the respiratory compensation point. In controls, RCP preceded a statistically significant increase in plasma [ATP]. Therefore, it seems that the mechanism responsible for the moment of plasma ATP outflow is a variable that is independent of training type, and irrelevant to the training status (competitive athletes vs. controls). However, it affected the magnitude of the exercise response. Programmed training resulted in a much higher plasma [ATP] during exercise including maximum effort, contrary to the effects of recreational activity where the exercise-induced [ATP] increase was poorly visible. Furthermore, the annual changes in plasma [ADP] and [AMP] reflected the changes in [ATP] as its degradation products.

Exercise training has been shown to lower blood flow to the exercising leg at a given submaximal power output. Training adaptations lead to increased capillarization, optimized blood flow distribution, and higher O_2_ extraction within skeletal muscle [25,26]. Additionally, previous studies have shown an enhanced vasodilatory capacity in endurance-trained athletes during maximal effort [27,28]. We presumed that increased [ATP] has to occur to provide a comparable vasodilation effect and O_2_ delivery allowing for enhanced energy production from oxidative metabolism. Increased [ATP] in speed–power compared to endurance athletes during the exercise test was likely caused by a more rapid increase in anaerobic–aerobic metabolism ratio. We concluded that the relationship between [ATP] and the percentage ratio of low-to-high exercise intensity is altered in highly trained athletes. Vasodilation during exercise may require higher [ATP] to cover muscle demands during more intense efforts. However, during maximal and supramaximal whole-body exercise, cardiac function limitation and muscle vasoconstriction contribute to the incapability of the circulatory system to meet the increasing skeletal muscle metabolic demands [29]. Collectively, these observations suggest an inability to meet the increased metabolic demands during intense whole-body exercise. Nonetheless, it would be reasonable to suggest that well-trained subjects, due to specific training stimuli, can more effectively and precisely match blood flow and O_2_ delivery with demand, and as a result, delay the inability to cover the metabolic requirements of the skeletal muscle. As mentioned above, this specific feature may depend on the predominant metabolism type in different sport disciplines. Another reason might be that the structure of training loads brings about specific adaptations [30]. We assume that short sprint bouts (predominance of anaerobic metabolism), dominant in sprinters, accounted for a pronounced increase in plasma [ATP] during exercise. In futsal players, [ATP] also increased in consecutive phases of the annual training cycle around the exercise test. However, this was most likely attributed to the demands of high-intensity intermittent efforts and a large number of matches and training sessions during the spring and autumn round in the competition period. In endurance athletes, a much higher volume of training (number of sessions and total net time) in the low-intensity and moderate-intensity energetic zone could result in lower needed plasma [ATP].

In trained individuals, there might be a greater increase in the arterial–venous O_2_ difference [15], which suggests enhanced O_2_ extraction in the active muscle capillary beds. Although ATP is released from the erythrocytes together with the O_2_ off-loading [5], training adaptations may to some extent explain enhanced [ATP] among highly trained athletes compared to controls. This emphasizes the training status as a significant variable. The differences between [ATP] curves around exercise over the annual training cycle also indicated that endurance, speed–power, and mixed training had a comparable effect on vascular response during exercise. However, the absolute maximal [ATP] was different between competitive athletes depending on sports discipline. Selection for a particular sport and predispositions to specific efforts may be relevant. Specific requirements of a training type result in the magnitude of response to an exhaustive treadmill test. The reasons for such discrepancies are unknown, but could be related to differences in exercise type (endurance vs. resistance training). Physical activities such as futsal and sprinting encompass both endurance and speed–power components, whereas triathlon/endurance athletes predominantly rely on aerobic energy sources. Further studies will be needed to better understand training-induced vascular function adaptations in highly trained athletes.

It has been proposed that inadequate cardiac output and peripheral vasoconstriction substantially limit skeletal muscle blood flow during severe whole-body exercise, despite increased peripheral blood flow and O_2_ demand [29]. The sympathetic nervous system is strongly involved in local vasoconstriction. It limits blood flow once a certain point is reached, which indicates that metabolic vasodilatation does not override sympathetic vasoconstriction activity in intense whole-body exercise [29]. In our study, exercise [ATP] sharply increased when metabolic demand started to be increasingly covered by anaerobic sources (85–95% VO_2_max). Attenuated vasodilatory activity due to increased contribution of anaerobic metabolism during incremental whole-body exercise may explain the diverse ATP response. Shepherd et al. [9] observed significant additional vasodilatation during exercise with simultaneous exogenous ATP infusion. This suggests that other substances are responsible for dilatation and/or additional endogenous ATP is released during exercise, causing further vasodilatation. This is in line with our results that showed that sports disciplines containing greater high-intensity training loads (sprinters and futsal players) required enhanced plasma [ATP] during exercise. Furthermore, a year-long cycle resulted in increased exercise and post-exercise [ATP] in all competitive athletes. An increase in [ATP] during exercise may result in additional vasodilatation to meet the increased blood flow and O_2_ demand. Previous research has shown that [ATP] increased in proportion to workload at higher intensities [2,31]. Considering all the above, we presume that the magnitude of [ATP] increase during exercise can be modulated by structured training, especially when high-intensity load predominates.

It has been suggested that high-intensity training leads to a reduction in the α-adrenergic responsiveness and improves functional sympatholysis at rest [32,33]. Kruse et al. [34] concluded that faster compared to slower volume-matched muscle contractions led to improved functional sympatholysis muscle contractions in humans. The question is whether increased [ATP] during exercise in consecutive phases of a one-year training cycle affects the ability to override sympathetic vasoconstriction, or simply that larger [ATP] is required for an adequate response to exercise. It has been demonstrated that intravascular [ATP] draining active skeletal muscle increases progressively with exercise intensity in young healthy adults [2,31], and has an intensity-dependent ability to limit α_1_-mediated vasoconstriction [8]. Importantly, training-induced lowering of the α-adrenergic responsiveness in humans [32] facilitates the increases in muscle blood flow in trained leg muscles during exercise. However, exercise training reduces the vasodilatory response to arterially infused ATP, suggesting that physical activity may alter purinergic P_2_ receptor sensitivity and/or ATP degradation in plasma [32,35]. Based on our results showing increased exercise and post-exercise [ATP] during an annual training cycle, we assume that the type of training may influence the physiological mechanisms of ATP release and/or degradation and its influence on muscle vessel dilatation during incremental exercise. However, the effect of programmed specific exercise training on intravascular ATP signaling, and thus on sympatholytic capacity, especially in highly trained individuals, needs to be investigated.

## 4. Materials and Methods

### 4.1. Subjects

Thirty-three highly trained male athletes from different sporting disciplines were studied in the Human Movement Laboratory at the Poznan University of Physical Education. Eleven male sprinters aged 24.1 ± 3.3 y, body height 186.2 ± 4.6 cm, having practiced competitive sport for 8.6 ± 2.3 y, and with a maximum heart rate (HR_max_) of 189 ± 9 beat/min, participated in the study. Eleven male endurance athletes (long-distance runners and triathletes) and 11 male futsal players aged 23.3 ± 4.1 y and 25.8 ± 4.0 y, body height 182.0 ± 5.6 cm and 181.3 ± 6.1 cm, having practiced competitive sport for 8.5 ± 1.9 y and 10.1 ± 3.9 y, and with a HR_max_ of 192 ± 7 and 187 ± 11 beat/min, respectively, participated in the study. All athletes competed at the international and Olympic level. The control group consisted of 11 healthy male recreationally active runners aged 27.5 ± 3.8 y, body height 180.0 ± 5.6 cm, without previous and current competitive sports experience, HR_max_ 189 ± 8 beat/min. All participants were healthy during the whole study period, having all hematological variables in the normal range. More detailed descriptive and exercise characteristics are presented in Table 2.

### 4.2. Study Design

An incremental running treadmill test until voluntary exhaustion, as described below, was used to assess the changes in exercise and post-exercise variables between training subphases. For all subjects, the same criteria for achieving maximal values were established. Each testing session was preceded by two days of reduced training volume and intensity. The study procedure was adapted to the training phases of the annual cycle: the first measurement was performed after the transition phase, second after the general subphase, third after the specific subphase of the preparatory phase, and the fourth, final examination was performed before the tapering period during the competition phase. Biochemical parameters were measured at rest, 4–5 times during the incremental exercise, and up to 30 min after exercise. During the incremental test, cardio-respiratory characteristics were monitored. All procedures and potential risks were explained and informed consent was obtained from each participant. The study was approved by the Local Bioethical Committee at the Karol Marcinkowski Poznan University of Medical Sciences. During all examinations, the ambient temperature remained unchanged at 20–21 °C.

### 4.3. Somatic and Physiological Variables

Weight and height were measured using a digital stadiometer (SECA 285, SECA, Hamburg, Germany). Body composition evaluation was performed using the dual X-ray absorptiometry method (DXA; Lunar Prodigy device; GE Lunar Healthcare, Madison, WI, USA) and analyzed using enCORE 16 SP1 software. All DXA scans were performed and analyzed following the best practice protocol proposed by Nana et al. [36]. Total-body skeletal muscle mass was calculated according to Kim et al. [37]. Heart rate was measured with Polar Bluetooth Smart H6 monitors (Polar Electro Oy, Kempele, Finland). An incremental running test (H/P Cosmos Pulsar, Sports & Medical, Nussdorf-Traunstein, Germany) was performed after 3 min of standing on the treadmill, the initial speed was 4 km·h^−1^ for the first 3 min, then increased to 8 km·h^−1^ and increased by 2 km·h^−1^ every 3 min until volitional exhaustion. VO_2_max was considered to be achieved if the test met at least three of the following criteria: (i) a plateau in VO_2_ despite an increase in workload; (ii) cutoff blood lactate concentration ≥9 mmol·L^−1^; (iii) RER ≥ 1.10; and (iv) heart rate ≥95% of the age-predicted HRmax [38]. The respiratory compensation point was determined based on the breaking point in the VE/VO_2_ and VE/VCO_2_ curve [39]. Athletes had their VO_2_max and main cardiorespiratory variables determined using MetaMax 3BR2 ergospirometer and analyzed by MetaSoft Studio 5.1.0 Software (Cortex Biophysik, Leipzig, Germany).

### 4.4. Hematological and Lactic Acid Measurements

Blood samples for hematological parameters were carried out as described elsewhere [40]. For lactic acid measurement, lithium heparin was used as an anticoagulant (S-monovette, 2.7 mL KE, Sarstedt, Nümbrecht, Germany). Lactate in whole blood (20 µL) was immediately assayed using the spectrophotometric enzymatic method (Biosen C-line, EKF Diagnostics, Barleben, Germany).

### 4.5. Plasma Nucleotide Measurements

Plasma nucleotide concentration analyses were performed using high-performance liquid chromatography (HPLC) with UV detection, according to the methodology of Smolenski et al. [41]. The catheter (1.3 × 32 mm, BD Venflon Pro, Becton Dickinson, Helsingborg, Sweden) was placed into the antecubital vein. Blood samples (2 mL) were drawn at rest, during, and after exercise using syringes containing ethylenediamine tetraacetic acid (EDTA) (S-monovette, 2.7 mL KE, Sarstedt, Nümbrecht, Germany). Samples were immediately centrifuged (Universal 320R, Hettich Lab Technology, Tuttlingen, Germany) for 30 s at 14.000 rpm in 4 °C. Subsequently, 200 µL of plasma was frozen down in liquid nitrogen in duplicate and stored at −80 °C until analysis. Samples were extracted using perchloric acid (2.4 mol·L^−1^) on ice at the ratio of 1:0.25 for 15 min and then centrifuged at 13.000 rpm for 3 min in 4 °C. The collected supernatant was neutralized using 3 mol·L^−1^ K_3_PO_4_ and centrifuged at 13.000 rpm for 3 min in 4 °C. The samples were left on ice for 30 min to ensure complete precipitation of potassium perchlorate. Then, supernatants were collected and stored at −80 °C before analysis. The analyses were performed using a Specta HPLC system (Thermo Fisher Scientific, Waltham, MA, USA) equipped with a 10 cm path flow cell to increase sensitivity. The separation was achieved with an analytical column BDS Hypersil C18 (150 mm × 4.6 mm × 3 μm; Thermo, Waltham, MA, USA) placed in a thermostat (18 °C) supported by a precolumn 20 mm × 4 mm (Phenomenex, type SecurityGuard, Torrance, CA, USA). The mobile phase consisted of A: 122 mM KH_2_PO_4_, 150 mM KCL, and 28 mM K_2_HPO_4_ and B: 15% (*v*/*v*) acetonitrile in A. The percentage of B changed from 0% to 100% in several linear steps during analysis and then returned to 0% B for equilibration. The whole separation time was 13.5 min and was conducted at 0.9 mL·min^−1^ flow rate with a sample injection volume of 40 µL. The quantitative analyses were performed based on the external calibration of the signal at 254 nm. Data acquisition and processing were managed by the Xcalibur™ software (v. 2.1, Thermo Scientific™, Waltham, MA, USA). The above-described method provided coefficients of variation <5% at different ATP concentrations.

### 4.6. Statistical Analyses

A one-way repeated measures ANOVA was performed to assess the differences in measured variables between consecutive examinations and between measurement points during exercise and recovery within each group of participants. Furthermore, a one-way ANOVA was performed to estimate the differences in nucleotide concentrations between groups at maximal exercise in the same training phase. If a significant difference was found (*p* < 0.05), post hoc Scheffe tests were performed. The effect size for ANOVA analyses was small to large for descriptive characteristics (η^2^ = 0.01–0.68), and statistical power was 0.07–1.00. The effect sizes for ANOVA analyses for plasma [ATP], [ADP], and [AMP] were large within groups between measurement points (η^2^ = 0.84–0.98). The statistical power for ANOVA at α = 0.05 for plasma [ATP], [ADP], and [AMP] between measurement points was 1.00. The effect sizes for ANOVA at the same measuring point between four consecutive examinations were large for plasma [ATP] (η^2^ = 0.29–0.93), ADP (η^2^ = 0.24–0.94) and [AMP] (η^2^ = 0.24–0.92), except for the control group where effect sizes for ANOVA were small to large for plasma [ATP] (η^2^ = 0.02–0.14), [ADP] (η^2^ = 0.01–0.23), and [AMP] (η^2^ = 0.04–0.24). The statistical power for ANOVA at α = 0.05 for plasma [ATP], [ADP], and [AMP] between four consecutive examinations within competitive athletes were 0.66–1.00, except for controls (0.07–0.68). The effect size and statistical power for ANOVA between groups at maximal exercise in each training phase were 0.77–0.92 and 1.00, respectively. All calculations were performed using STATISTICA 13.1 software (StatSoft, Tulsa, OK). The significance level was set at *p* < 0.05. All values are presented as means ± SD.

## 5. Conclusions

In this study, we demonstrated that ATP concentration significantly changed over consecutive training phases in highly trained athletes in an annual training cycle. Sprint training brought about adaptations resulting in higher maximal exercise-induced plasma ATP levels compared to endurance and mixed training, and especially compared to recreational non-periodized activity. In spite of differences in magnitude, each kind of structured training program (sprint, endurance, or mixed) incorporating a sufficient amount of high-intensity exercise led to the same adaptation pattern. The key factor seems to be the proportion of high-intensity training loads that are related to an increased exercise-induced plasma [ATP] in the competition period, whereas the reduction or lack of high-intensity exercise in other training phases is associated with a decrease in plasma [ATP].

## Figures and Tables

**Table 1:** Typical structure of training loads in tested futsal players, endurance athletes, and sprinters in successive examinations in a 1 year cycle.

	2nd Examination *			3rd Examination **			4th Examination **		
	General Preparation			Specific Preparation ^†^			Competition Phase ^#^		
	FU	EN	SP	FU	EN	SP	FU	EN	SP
**Training sessions (no.)**	71	181/122	80	62	132/96	61	63	179/120	87
**Competitions (no.)**	10	−/−	−	11	4/5	6	13	6/9	8
**Net exercise time (hours)**									
total	84.3	225.5/151.3	92.6	70.1	201/142.4	67.3	70.4	212.2/140.3	100.1
per one training session	1.19	1.25/1.24	1.16	1.13	1.52/1.48	1.10	1.12	1.18/1.17	1.15
**Total training distance (km)**									
running	−	1975/−	−	−	501/−	−	−	589/−	−
swimming	−	251/−	−	−	162/−	−	−	204/−	−
cycling	−	865/−	−	−	2655/−	−	−	2875/−	−
**Exercise zones (% of total time)**									
low-intensity	74.3	83.9/83.6	70.6	67.4	81.3/80.9	82.5	67.5	78.0/75.6	73.3
moderate-intensity	18.3	14.4/14.5	19.7	19.2	13.8/14.1	7.1	19.6	11.8/13.9	23.0
high-intensity	7.4	1.7/1.9	9.7	13.4	4.9/5.0	10.4	12.9	10.2/10.5	3.7

Abbreviations: FU, futsal players; EN, endurance athletes (triathletes/long distance runners); SP, sprinters. * Data encompass the period between the beginning of the training cycle and the 2nd examination. ** Data encompass the period between the preceding and the present examination. ^†^ Spring round of the competitive season in futsal players. ^#^ Autumn round of the competitive season in futsal players.

**Table 2:** Descriptive and exercise characteristics in four consecutive training phases in futsal players (*n* = 11), endurance athletes (*n* = 11), sprinters (*n* = 11), and control group (*n* = 11).

	Transition	General	Specific	Competition	ANOVA *
**Body Mass (kg)**					
Futsal Players	75.8 ± 6.9	76.9 ± 7.0	77.6 ± 7.8 ^a^	77.9 ± 7.4 ^a^	0.006
Endurance	74.6 ± 8.1	73.1 ± 7.6 ^†^	73.7 ± 6.7 ^†^	73.2 ± 7.3 ^†^	0.078
Sprinters	81.6 ± 5.5	82.8 ± 5.4	82.7 ± 5.6	83.3 ± 6.1 ^a^	0.009
Control group	77.2 ± 7.9	77.8 ± 8.0	76.9 ± 7.6	76.8 ± 7.0	0.295
ANOVA **	0.125	0.022	0.039	0.014	
**Total-body SMM (kg)**					
Futsal Players	33.0 ± 3.0 ^†^	33.9 ± 3.6 ^†,a^	34.1 ± 3.3 ^†,a^	34.2 ± 3.2 ^†,a^	0.001
Endurance	32.9 ± 3.6 ^†^	32.8 ± 3.3 ^†^	33.1 ± 3.3 ^†^	33.0 ± 3.2 ^†^	0.672
Sprinters	39.1 ± 3.7	40.5 ± 3.6	40.4 ± 3.8	41.4 ± 4.6 ^a^	0.002
Control group	33.3 ± 3.1 ^†^	33.5 ± 3.2 ^†^	33.0 ± 3.6 ^†^	33.5 ± 3.6 ^†^	0.335
ANOVA **	0.000	0.000	0.000	0.000	
**Total-body fat (%)**					
Futsal Players	17.4 ± 3.0 ^†^	16.4 ± 2.1 ^†^	16.7 ± 2.7 ^†^	17.1 ± 2.3 ^†^	0.391
Endurance	16.1 ± 2.6	14.0 ± 2.7 ^a^	14.5 ± 2.3 ^†,a^	14.2 ± 2.1 ^†,a^	0.002
Sprinters	12.6 ± 2.2	11.0 ± 2.0 ^a^	10.8 ± 1.9 ^a^	10.6 ± 1.8 ^a^	0.000
Control group	18.4 ± 3.9 ^†^	18.5 ± 4.2 ^‡,†^	18.2 ± 3.7 ^‡,†^	17.2 ± 4.2 ^†^	0.315
ANOVA **	0.000	0.000	0.000	0.000	
**LA_rest_ (mmol·L^−1^)**					
Futsal Players	1.4 ± 0.4	1.2 ± 0.2	1.0 ± 0.2 ^a^	0.8 ± 0.2 ^a,b^	0.000
Endurance	1.2 ± 0.3	1.0 ± 0.2	1.0 ± 0.2	0.9 ± 0.1 ^a^	0.006
Sprinters	1.4 ± 0.6	1.4 ± 0.5	1.2 ± 0.4	0.9 ± 0.2	0.023
Control group	1.3 ± 0.3	1.4 ± 0.3	1.2 ± 0.3	1.1 ± 0.2 ^§,‡,b^	0.015
ANOVA **	0.652	0.033	0.057	0.002	
**LA_max_ (mmol·L^−1^)**					
Futsal Players	11.6 ± 2.2	11.2 ± 2.9	10.8 ± 2.4	9.9 ± 1.5	0.065
Endurance	11.2 ± 1.8	9.9 ± 2.1	10.2 ± 1.9	10.1 ± 1.5	0.135
Sprinters	10.7 ± 1.9	10.8 ± 2.2	9.6 ± 1.9	10.0 ± 1.4	0.026
Control group	10.7 ± 1.4 ^b^	11.6 ± 1.8	10.2 ± 1.9 ^b^	10.6 ± 2.1 ^b^	0.001
ANOVA **	0.543	0.319	0.575	0.784	
**VO_2_max (ml·kg^−1^·min^−1^)**					
Futsal Players	55.81 ± 3.94 ^‡^	55.57 ± 2.81 ^‡^	57.04 ± 2.18	58.47 ± 2.06 ^‡,†^	0.063
Endurance	64.58 ± 3.52	65.26 ± 7.81	67.72 ± 3.15	66.81 ± 4.66	0.392
Sprinters	52.53 ± 4.32 ^‡^	53.01 ± 4.19 ^‡^	52.88 ± 3.92 ^‡^	52.91 ± 3.92 ^‡^	0.932
Control group	57.92 ± 3.42 ^‡,†^	57.38 ± 4.25 ^‡^	56.66 ± 3.08 ^‡^	55.96 ± 3.47 ^‡^	0.128
ANOVA **	0.000	0.000	0.007	0.000	

Abbreviations: SMM, skeletal muscle mass; LA_rest_, resting lactate concentration; LA_max_, maximal lactate concentration; VO_2_max, maximal oxygen uptake. Values are means ± SD. * one-way ANOVA between examinations within group; ** one-way ANOVA between groups at the same examination period. ^§^ Significantly different from FU. ^‡^ Significantly different from EN. ^†^ Significantly different from SP. ^a^ Significantly different from transition phase. ^b^ Significantly different from general preparation phase. ^c^ Significantly different from specific preparation phase.
